# Becoming an Age-Friendly University: The Ongoing Journey of the University of Mississippi

**DOI:** 10.1093/geroni/igaf122.1002

**Published:** 2025-12-31

**Authors:** Keith Anderson

**Affiliations:** University of Mississippi, Oxford, Mississippi, United States

## Abstract

The University of Mississippi (UM) joined the Age-Friendly University (AFU) Global Network in 2024, approximately two years after this GSA Fellow arrived on campus. The first step in the process was to educate key personnel on the meaning and importance of being an AFU. We quickly learned that most university and community members did not fully understand the concept, but there was genuine interest. We then formed a committee and inventoried age-related initiatives. We found that programs and resources existed (e.g., gerontology courses, older adult learning programs); however, utilization was often limited due to format (e.g., in-person, online), access (e.g., parking, cost), and lack of awareness. In the final step in the application process, we identified strengths and, equally important, deficits and unmet opportunities. While our efforts to join the AFU Global Network were ultimately successful, much work is left to be done. UM and Mississippi are unique in many ways. UM is a large, R1 university located in a rural and diversly populated state. Building a network of age-friendly organizations, including those that serve diverse populations (e.g., HBCUs, rural health departments) is key to reaching all older adults across the state. UM’s main campus is located over two hours away from UM’s Medical Center, a major resource for age-related programming, education, and research. Closing this distance through effective communication and collaboration is essential. Finally, embedding the AFU concepts into the fiber of UM and Mississippi, rather than in a few champions, will ensure continued progress toward our age-friendly goals.

